# Review of therapeutic agents for burns pruritus and protocols for management in adult and paediatric patients using the GRADE classification

**DOI:** 10.4103/0970-0358.70721

**Published:** 2010-09

**Authors:** Ioannis Goutos, Maria Clarke, Clara Upson, Patricia M. Richardson, Sudip J. Ghosh

**Affiliations:** Department of Plastic Surgery, Queen Alexandra Hospital, QUAD Building, Southwick Hill Road, Cosham, Portsmouth, PO6 3LY, United Kingdom; 1 Departments of Occupational Therapy and Physiotherapy, Stoke Mandeville Hospital, Mandeville Road, Buckinghamshire, HP21 8AL, United Kingdom; 2 Department of Anaesthesia, St. Andrew’s Centre for Plastic Surgery and Burns, East Wing, Broomfield Hospital, Court Road, Chelmsford, CM1 7ET, United Kingdom; 3 Department of Plastic Surgery, Stoke Mandeville Hospital, Mandeville Road, Buckinghamshire, HP21 8AL, United Kingdom

**Keywords:** Pruritus, burn, itch, antihistamines, gabapentin

## Abstract

To review the current evidence on therapeutic agents for burns pruritus and use the Grading of Recommendations, Assessment, Development and Evaluation (GRADE) classification to propose therapeutic protocols for adult and paediatric patients. All published interventions for burns pruritus were analysed by a multidisciplinary panel of burns specialists following the GRADE classification to rate individual agents. Following the collation of results and panel discussion, consensus protocols are presented. Twenty-three studies appraising therapeutic agents in the burns literature were identified. The majority of these studies (16 out of 23) are of an observational nature, making an evidence-based approach to defining optimal therapy not feasible. Our multidisciplinary approach employing the GRADE classification recommends the use of antihistamines (cetirizine and cimetidine) and gabapentin as the first-line pharmacological agents for both adult and paediatric patients. Ondansetron and loratadine are the second-line medications in our protocols. We additionally recommend a variety of non-pharmacological adjuncts for the perusal of clinicians in order to maximise symptomatic relief in patients troubled with postburn itch. Most studies in the subject area lack sufficient statistical power to dictate a ‘gold standard’ treatment agent for burns itch. We encourage clinicians to employ the GRADE system in order to delineate the most appropriate therapeutic approach for burns pruritus until further research elucidates the most efficacious interventions. This widely adopted classification empowers burns clinicians to tailor therapeutic regimens according to current evidence, patient values, risks and resource considerations in different medical environments.

## INTRODUCTION

Pruritus is a common and distressing symptom affecting the majority of burns patients’ rehabilitation. Recent work has confirmed the lack of a unified approach in assessing and managing the symptom in UK burns units.[1] One of the major reasons impeding an evidencebased approach is the paucity of high-quality studies evaluating the effectiveness of different agents for burns pruritus and the absence of a systematic appraisal of therapeutic protocols. We present a review of all therapeutic approaches in the burns literature and explore the utility of the Grading of Recommendations Assessment, Development and Evaluation (GRADE) classification to suggest a template for management in adult and paediatric patients.

## BACKGROUND

Pruritic stimuli are transmitted by a subpopulation of C-fibres extending from the skin to the dorsal root ganglion of the spinal cord. Subsequently, impulses are conveyed onto higher central nervous system centres to reach the cortical areas, including the somatosensory, motor, prefrontal cortical and cingulated gyral areas.[2–8] The two parts of the pruritic pathway, namely the peripheral and the central, form different targets for pharmacological intervention.

A small number of protocols for burns pruritus have been published in the literature. One uses oral antihistamines and topical emollients as first line, followed by the recruitment of a clinical psychologist and a variety of third-line agents, including capcaisin, transcutaneous electrical nerve stimulation (TENS), massage and silicone sheets.

The hierarchy of this therapeutic algorithm is largely based on the risk of adverse effects, which increase from the first to the last step of the protocol.[9] One of the major limitations of this work is the lack of a standardised methodology to appraise the studies in the field and the heavy reliance on the risk of sideeffects as a criterion to prioritise therapeutic agents. A second group used the Physiotherapy Evidence Database (PEDro) scale to stratify studies and concluded with a treatment template, which recommends moisturisation and massage as the starting point of therapy and then offers a variety of options to clinicians for burns patients according to the total burn surface area (TBSA).[10] The inherent weakness in this approach is that it relies on a pure evidence-based approach to draw guidelines in a subject area characterised by a profound lack of highquality studies.

Since the 1970s, a growing number of organisations have used various different systems to grade the quality of evidence and stratify the strength of recommendations in medical practice.

The most popular techniques described in the literature are the Delphi method, the nominal group technique and a combination of these two approaches.[11] The Delphi method involves experts submitting opinions independently and revising them following the study of other expert opinions provided anonymously by a facilitator. The process concludes with convergence into a common answer to the study question.[12] The nominal technique draws upon opinions from a small number of experts, who interact in person with a view to coming to a conclusion on a certain topic.[11] Previous grading systems employ up to nine categories of recommendations, with symbols being either numbers or letters. As a result of this heterogeneity, communication between specialists regarding different guidelines can be confusing.[13]

The GRADE working group began as an informal collaboration of individuals with an interest in tackling the shortcomings of previous grading systems. The principles governing the system allow for more consistent judgements and communication by using two discrete categories of recommendations; strong and weak. Key factors determining the strength of recommendation are[14]:

- The balance between desirable and undesirable consequences of a particular and alternative management strategies.
- The quality of evidence, with randomized controlled trial results carrying more weight than observational studies.
- Patient values and preferences variability.
- Cost/resource allocation. This is variable over time and geographical location.

It is evident that quality of evidence is only one of the factors implicated in the decision about whether a particular intervention will be recommended strongly or weakly by members of the panel. A strong recommendation reflects the consensus judgement that the desirable effects of an intervention outweigh the undesirable effects (and allows the evidence deriving from observational studies to be upgraded as high quality and vice versa for highquality studies). A weak recommendation implies that the desirable effects will outweigh the undesirable effects but the panel is not confident about the tradeoffs, either because key evidence is of low quality or because the benefits and drawbacks are closely balanced.[11] The approach to decide on the weight of different factors is subjective and calls upon the opinions of a variety of healthcare professionals. Given the paucity of randomised controlled trials for burns pruritus, it is impossible to assess whether results from low-statistical quality studies will predict results of future higher quality trials. Conversely, randomised trials may not always reflect the effect on the majority of the patients because only highly selected and motivated individuals relative to the interest population will participate (concept of ‘directness’).

The Surviving Sepsis Campaign employed a GRADE approach to classify the quality of evidence regarding managing severe sepsis and septic shock, recruiting 50 experts from more than 10 countries.[15]

Since 2006, the British Medical Journal has requested in its “instructions to authors” that authors should preferably use the GRADE system for appraising evidence when submitting a clinical guidelines article. Additionally, the World Health Organization and the Cochrane Collaboration are among the 25 organisations that have adopted this classification.[16] The advantages of using the GRADE system include:

- Clear separation of quality of evidence and recommendation.
- Explicit, comprehensive criteria for downgrading and upgrading quality of evidence ratings and acknowledgement of patient values and preferences.
- A transparent process of moving from evidence to recommendations.

Disadvantages of the GRADE classification include a degree of arbitrariness in the discrete categorisation of quality of evidence and recommendation strength with both being a continuum.[16]

## MATERIALS AND METHODS

We adopted the following methodology to appraise the literature and derive our multidisciplinary protocols

**Figure 1:**
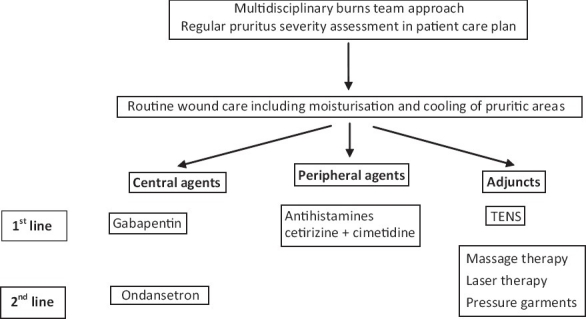
Grading of recommendations, assessment, development and evaluation-based protocol for the management of burns pruritus in adult patients

**Figure 2:**
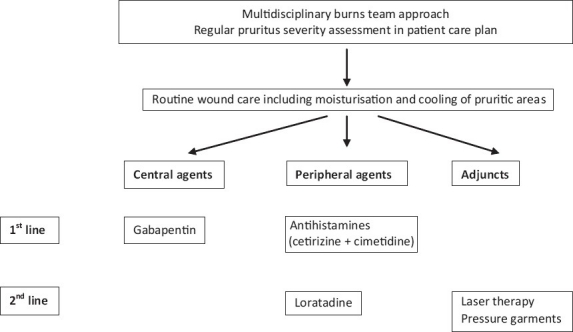
Grading of recommendations, assessment, development and evaluation-based protocol for the management of burns pruritus in paediatric patients

- Identification of the evidence. Our assigned librarian conducted a comprehensive literature search using the Cochrane Database of systematic reviews, MEDLINE and Embase between 1966 and the present time. We also collected data from internet journal sources and abstracts from relevant conferences in an attempt to be exhaustive in our search.
  We categorised papers on burns pruritus in terms of levels of evidence using the NHS Centre for Reviews and Dissemination (CRD) as shown below.[17]
  Level 1: Experimental studies (randomised controlled trials with concealed allocation).
  Level 2: Quasi-experimental studies (experimental studies without randomisation).
  Level 3: Controlled observational studies.
    3a: Cohort studies.
    3b: Case–control studies.
  Level 4: Observational studies without control groups.
  Level 5: Expert opinion based on pathophysiology, bench research or consensus.
- Formation of the multidisciplinary panel. The first author and panel coordinator (IG), who has an active ongoing interest in the pathophysiology and treatment of burns pruritus, invited the following experts to appraise the studies individually using the GRADE system:
  MC is a senior occupational therapist with 27 years of experience in acute and reconstructive burns care.
  CU is a senior physiotherapist with 8 years of experience in burns scar management.
  PMR is an anaesthesiology consultant with 10 years service in a regional burns centre and has an ongoing interest in the management of burns pain and itch.
  SJG is a consultant in burns surgery with 10 years of experience in acute burns care provision and rehabilitation.
- Presentation of evidence. Three separate tables were compiled summarising the salient points of relevant papers identified for adult, paediatric and mixed (adult and paediatric) patients [Tables 1, 2, 3]. These tables include the type of intervention evaluated, the CRD level of evidence 1-5, number of included patients, the study design outline and outcomes. Other data incorporated include any reported sideeffects and cost of the interventions. The latter is challenging to estimate given the different nature of the agents studied. The consensus decision of the panel was to calculate the cost of 1 month’s course of oral medication at the maximum dose as specified in the British National Formulary.[40] As far as topical treatments are concerned, we present the cost for 1 month of topical application for a 5% burn injury.
- Grading the strength of recommendation. All members of the panel were presented with the data for all eligible studies and were given scoring sheets, which comprised of four choices of scores for each agent as shown below.
  Score+1: strong recommendation in favour of an intervention.
  Score+2: weak recommendation in favour of an intervention.
  Score-2: weak recommendation against an intervention.
  Score-1: strong recommendation against an intervention.
  Each panel member assigned a particular grade to each study using the GRADE system and was encouraged to review the final individual scoring before submission to the panel coordinator. Subsequently the median score for each intervention was calculated to stratify the strength of the panel’s recommendation for individual agents (last column in Tables 1–3).
- Finalisation of the recommendations. Following collation of individual results, the panel coordinator drafted an integrated protocol for adult and paediatric patients separately reflecting the consensus views of the panel members (studies with a median score of +1 were included as first-line agents and those with a score of +2 as second line). The preliminary results were distributed to all panel members for final comments and adjustments before an agreement for the final version of the protocols was reached [Figures 1 and 2].

**Table 1:** Studies of interventions for burns pruritus in adult patients depicting agent/author of study, CRD level of evidence, salient design details, outcomes, reported side-effects/contraindications, cost (1 month treatment for maximum oral dose or a 5% injury for topical agents) and median GRADE score

*Author/agent*	*CRD level of evidence*	*Patient sample*	*Study design*	*Outcome*	*Side-effects/comments*	*Cost*	*Median grade score*
Vitale *et al*.[2]/antihistamines	Prospective cross-over observational study (4)	*N* = 40; average age: 35.9 ± 12.8 y	3 agents changed monthly in random order (hydroxyzine, chlorpheniramine, diphenhydramine); lubricants prn	20% pts reported complete relief, 60% partial, 20% of pts had no relief; no differences in agents tested; 61% pts preferred hydroxyzine, 26% chlorpheniramine, 13% diphenhydramine	C: 35 pts analysed (5 abandoned study due to itch cessation); 37% of respondents developed tolerance and dose increase restored response in 85% pts	£3.36.48	-2
Choiniere *et al*.[18]/capsaicin	Experimental study (1)	*N* = 30; mean age: 37.4 ± 11.9 y	0.025% caps vs. placebo fourtimes/ day for 6 weeks	No significant difference between the 2 groups	S/E: transient burning at application site	£184.80	-1
Hettrick *et al*.[19]/transcutaneous electrical nerve stimulation (TENS)	Experimental study (1)	*N* = 30	Control: pressure garments, skin lubrication, soft tissue mobilisation, PT/OT, antiitch medication; TENS group: 1 h/ day for 3 weeks over pruritic area (<150 μs, >180 Hz, low– comfortable level)	Change between pre- and post-TENS itch VAS significant (*P* = 0.086)	C/I: electrical injuries, pregnancy, epilepsy history, pacemaker *in situ*; C: 20 pts completed study; 10 withdrew/did not comply with study	£34, but nondisposable parts can be used sequentially by many patients	+1
Field *et al*.[20]/ massage therapy	Experimental study (1)	*N* = 20; average age = 38.2 y	Massage group: 30 min cocoa butter over a moderatesized area (trained therapists) twice/week for 5 weeks; control group: medical care, PT/OT cocoa butter application	Immediate reduction in itch following first and last sessions (*P* < 0.001; *P* < 0.005), improved pain, anxiety/mood ratings with massage		£120	+2
Whitaker[21]/ TENS	Case report (4)	*N* = 1 (19 y)	TENS over itchy area, 5–8 mA for 5 days before and 2 weeks after discharge	TENS stopped after 2 weeks, since patient’s symptoms improved significantly		£34, but disposable parts can be used sequentially by many patients	+2
Gaida *et al*.[22]/ low-lever laser	Case– control (3a)	*N* = 19; average age = 38 ± 13.97, range = 18–77 y	400 mW 670 nm Softlaser twice/week for 8 weeks, control area on each pt	Relief of itch in all pts (VAS drop from 4.36 ± 3.26 to 1.31 ± 1.88)		£50 (once laser equipment is available in the medical setting)	+2
Roh *et al*.[23]/skin rehabilitation massage	Pre test–post test study (3b)	*N* = 35; mean age = 39.1 ± 8.2 y	Massage group: 30 min/week by certified nurses + 10 min/day by caregiver for 3 months	Significant decrease in pruritus with massage than control (t =−2.942, *P* = 0.006)		£120	+2
Demling *et al*.[25]/Doxepin	Case–control study (3b)	*N* = 41	Doxepin group: cream qds + skin moisturiser 20 min later; control group: skin moisturiser + diphenhydramine + hydroxyzine (3-month study)	Significant reduction in itch and erythema for study length period; itch stopped in 55% pts before the end of the 3-month period vs. 10% in the oral medication group (response seen within 15 min of application)	S/E: mild and transient somnolence (15% doxepin vs. 80% standard care group), localised skin reaction in 1 patient (removed from the study)	£218.40	-1
Bauling *et al*.[26]/ dapsone	Observational study (4)	*N* = 8	Gel topically up to qds for 14 days	5 pts had significant relief, 2 moderate and 1 patient reported no symptomatic relief	C: No toxic serum dapsone levels recorded	£256.15	-2
LaSalle *et al*.[27]/ naltre-xone	Observational (4)	*N* = 13; average age = 43.3 ± 16.9 y, range = 19–78 y	50 mg/day naltrexone, antihistamines and hydrating lotion in addition to traditional therapy (1 pt required 100 mg naltrexone/ day)	72% satisfied with itch relief, 69% were able to reduce/ stop taking other medications to control itch, 85% recommended to other burn pts, 62% reported improved quality of life	C/I: abnormal LFT, opioid taking; C: 2 dropouts – 1 intolerable dizziness, 1 allergic reaction to dye in pills	£23.80	-2
So *et al*.[28]/ silicone sheeting	Experimental study (1)	*N* = 28	CEG group: routine product instructions (verbal and handout); EEG group: as above and additionally a 5-page handout and 26 min videotape	EEG group: steady and progressive decline in itching severity over 6 months of follow-up compared to the CEG group (*P* = 0.01)	C/I: inability to communicate in English, cognitive impairment, use of alternative scar treatments, open/ unstable wounds, facial scars; C: 3 pts lost to follow-up	£49.92	-2

N, number of patients; M, months; Y, years; prn, as required; pts, patients; C, comments; S/E, side–effects; PT, physiotherapy; OT, occupational therapy; C/I, contraindications; VAS, visual analogue scale; CEG, conventional education group; EEG, enhanced education group

**Table 2:** Studies of interventions for burns pruritus in paediatric patients depicting: agent/author of study, CRD level of evidence, study design details, outcomes, reported side-effects, cost (1 month treatment for maximum oral dose or a 5% injury for topical agents) and median GRADE score

*Author/agent*	*CRD level of evidence*	*Patient sample*	*Study design*	*Outcome*	*Side-effects/comments*	*Cost*	*Median grade score*
Mendham[29]/ gabapentin	Observational study (4)	*N* = 35; age: 6 m–15 y (weight: 10–60 kg	Unresponsive patient to chlorpheniramine + trimeprazine, dose: 5 mg/kg tds and increased as needed (max 10 mg/kg/day)	Marked response within 24 h with antihistamine reduction or discontinuation. Some pts stopped at 4 weeks, some continued up to 18 m, especially if hypertrophied scar present	C: 3 pts developed behavioural problems (2 responded to dose reduction, 1 with ADHD had to stop despite good itch control)	£26.04	-2
Kopecky *et al*.[30]/EMLA	Observational study (4)	*N* = 5; age: 1–5 y	Day 1 + 2: control; Day 3: EMLA applied for 1 hour on max skin surface area 600 cm^2^ if 10–19 kg and 2 hour or max 2000 cm^2^ if >20 kg, then removed; hydroxyzine prn for all patients	Mean number of pruritic episodes and antihistamine use greater on Day 1 and 2 than on Day 3 (*P* = 0.01; *P* = 0.03); blood levels nontoxic, no hypoxia reported, metHb = 1–3%		£273.00	-2
Barone *et al*.[31]/Unna boot	Observational study (4)	*N* = 6; age: 17–20 m	Gr 1: Unna boot, no antihistamines, weekly dressing change; Gr 2: conventional dressings, antihistamines, daily dressing changes	Dressing change duration 15 min vs. 3.5 h/week, cost $19.80 vs. $30.99, itch less troublesome, no systemic drugs needed, better appetite, sleep, play pattern with Unna boot		£48.44	-1
Tager *et al*.[32]/ Loratadine	Observational study (4)	*N* = 32; age: 2 m–15.8 y	Unresponsive to diphenhydramine and hydroxyzine	Subjective relief from itching in all patients		£0.84	+2

N, number of patients; m, months; y, years; C, comments; prn, as required; ADHD, attention deficit hyperactivity disorder; metHb, methaemoglobin

**Table 3:** Studies of interventions for burns pruritus in mixed (adult and paediatric) patients depicting: agent/author of study, CRD level of evidence, study design details, outcomes, reported side-effects/contraindications, cost (1-month treatment for maximum oral dose or a 5% injury for topical agents) and median GRADE score

*Author/agent*	*CRD level of evidence*	*Patient sample*	*Study design*	*Outcome*	*Side-effects/comments*	*Cost*	*Median grade score*
Baker *et al*.[33]/ antihistamines	Doubleblinded, crossover, placebocontrolled (1)	*N* = 32 (only 17 completed study by adhering to protocol); age range = 10–60 y, mean = 35.7 y	16 days divided in 4-day intervals, cetirizine + cimetidine vs. diphenhydramine + placebo	Cetirizine + cimetidine group: dramatic improvement at 1 + 6 h and moderate impact at 12 h after initial medication compared tothe diphenhydramine/ placebo group	S/E: drowsiness, dry mouth, headache	£4.48	+1
Matheson *et al*.[34]/colloidal oatmeal	Cohort study (2)	*N* = 35; age range = 14–64	5% colloidal oatmeal + liquid paraffin vs. liquid paraffin bath and moisturiser (methdilazine 8 mg tds prn)	Significant difference in reported daily itch and decrease in antihistamine usage in the colloidal oatmeal group (*P* < 0.001)	C: neither product completely relieved itch in every patient who used them; 1 pt dropped out due to delirium	£16.80	-2
Brooks *et al*.[35]/ acticoat	Case series (4)	*N* = 5	2-week application at 2, 3, 3, 6, 8 months postburn	Significant drop in itch VAS (*P* = 0.0022)		£150	-2
Allison *et al*.[36]/585 nm laser flashlamppumped pulsed dye laser	Case–control (3a)	*N* = 38; mean age = 33.3 ± 8.3 y	3 treatments at monthly intervals and assessment at 6 + 12 months, -585 nm, 5 mm diameter spot at 5–6 J/m2	Itch in both treated and control areas improved posttreatment (*P* < 0.0001) and remained improved at 6 + 12 months (*P* < 0.0001); improvement level greater in treatment areas compared with control at 1, 6, 12 months (*P* = 0.009, *P* = 0.024, *P* = 0.044)	S/E: stinging sensation at application site; C: 1 withdrawal due to scar breakdown	£50 once equipment is available in the medical setting	+2
Eldardiri *et al*.[37]/ antihistamines and gabapentin	Open trial (4)	*N* = 50	Cumulative approach with moisturisation (step 1) and stepwise introduction of chlorpheniramine (step 2), hydroxyzine and cyproheptadine (step 3), gabapentin (step 4)	Step 2 effective in 10% pts, step 3 polytherapy effective in 84%pts		£10.08 (step 2), £11.20 (step 3)	+1
Goutos *et al*.[38]/ gabapentin and antihistamines	Open trial (4)	*N* = 41	Cumulative approach with moisturisation (step 1) and stepwise introduction of gabapentin (step 2), cetirizine and cyproheptadine (step 3), chlorpheniramine (step 4)	Step 2 effective in 41.46% pts, step 3 polytherapy effective in 95.12% pts		£26.04 (step 2), £29.96 (step 3)	+1
Leung *et al*.[39]/ pressure garments	Observational (4)	*N* = 100	Lycranet garment, for an average of 10 months average with 6-monthly assessments (excellent to poor response scale)	Itch response extremely satisfactory; onset of itch relief almost instantaneous	S/E: occasional blistering, in some cases treatment had to be delayed/ stopped	£25	+2

(N, number of patients; y, years; S/E, side–effects; C, comments; prn, as required; PT, physiotherapy; OT, occupational therapy; C/I, contraindications; VAS, visual analogue scale)

## RESULTS

Analysis of the available literature in the subject of postburn itch reveals that the overwhelming majority of studies (16 out of 23) are of an observational design, which corresponds to CRD levels 3 and 4. Any attempt to rely on an evidence-based approach to derive therapeutic protocols is severely hindered by the lack of high-quality studies. The employment of the GRADE classification has the potential to dictate the best available agents for therapy by using four different parameters, with quality of evidence being one of these. We present the principles underlying our consensus treatment templates for adult and paediatric patients.

### Multidisciplinary approach

Pruritus is one of the many distressing symptoms that burns patients will experience during their rehabilitation. Successful assessment and treatment needs to be viewed in the context of a holistic multidisciplinary approach.

We advocate the early involvement of all members of the burns team, including surgeons, anaesthesiologists, physiotherapists, occupational therapists, nurses and psychologists. Each member of the team has a unique role to play in identifying and managing nociceptive symptoms and multidisciplinary meetings are crucial in coordinating therapeutic strategies for individual patients.

### Employment of a tool to assess severity of symptoms and response to treatment

Quantification of symptom severity is paramount in planning optimal therapeutic interventions and defining the clinical response. Recent work has confirmed that higher itch scores before treatment predict the need to recruit a combination of agents to achieve satisfactory relief from pruritic symptoms.[38] There is paucity of standardised tools for pruritic symptom evaluation in the burns literature. The ‘itch man scale’ is a versatile numerical rating scale combined with a pictorial element[41] that has been validated in paediatric patients.[42] Our clinical experience with this tool has been positive in both adult and paediatric patients and we recommend it for inclusion in daily care plans for pruritus assessment in burns patients.

### Simple wound care including moisturization and cooling of pruritic areas

The use of topical emollients and cooling agents to the pruritic wounds/scars is a widespread practice in burns units. There are no studies to support the use of these agents. Nevertheless, they form an integral part of skin care routine, and the globally positive clinical experience allows their inclusion in our protocols.

### Agents acting on the central part of the pruritic pathway

Gabapentin forms the first-line, centrally acting agent in both the adult and the paediatric protocols. A comparative study of two stepwise protocols in inpatient burns victims has revealed that gabapentin monotherapy is four-times more effective than chlorpheniramine monotherapy (t = 3.70, df = 89, *P* < 0.001).

Additionally, gabapentin in combination with two antihistamines (cetirizine, cyproheptadine) rendered a statistically significant higher proportion of patients itch-free than the following combination of three antihistamines – chlorpheniramine, hydroxyzine and cyproheptadine (χ^2^ = 12.2, df = 1, *P* = 0.001).[38]

Gabapentin has also been shown to have an opioidsparing effect on acutely burnt patients.[43] This appears to persist into the post-treatment period and has been attributed to an effect on nociceptive processes involving sensitisation in the central nervous system. It is possible that gabapentin is more effective than antihistamines by targeting the central nervous system components, where a variety of peripheral pruritic impulses (including those mediated by histamine) converge. It is also plausible that gabapentin may prevent the sensitisation of neuronal pathways leading to neuropathic mechanisms and the development of refractory pruritus.

Ondansetron forms the second-line, centrally acting agent in our adult protocol. No interventions focusing on the central nervous system pathway have been tested on paediatric patients, a fact that is reflected by the lack of a second-line, centrally acting agent in our paediatric protocol.

### Agents acting on the peripheral part of the pruritic pathway

Our protocols recommend the combination of cetirizine and cimetidine as the first-line choice of antihistamines for both adult and paediatric patients. The combination achieves comprehensive blockade of histaminic receptors employing a second-generation H1 partial agonist (cetirizine) and an H2 receptor antagonist (cimetidine).

H1 receptor antagonists are divided into first-generation antihistamines, which bind to muscarinic, alphaadrenergic and serotonergic as well as histaminic receptors, and second-generation antihistamines, which have minimal activity at the nonhistaminic receptors. The latter generation has a more favourable side-effect profile (including reduced sedation due to reduced central nervous system penetration) and a longer duration of action, necessitating less-frequent administration.[44]

Loratadine is another second-generation antihistamine that forms a second-line medication in our paediatric protocol.

### Non-pharmacological adjuncts

The use of TENS in adult patients is recommended as a first-line non-pharmacological intervention in our protocol. Second-line adjuncts include laser and pressure garment therapy in both adults and children and massage therapy in adult patients. Non-pharmacological adjuncts have a low level of uptake among UK burn units.[1] We believe that these adjuncts represent an effective alternative to pharmaceutical agents, especially in the quest to reduce polypharmacy in burns rehabilitation. Alternatively, they should be considered in combination with peripherally or centrally acting medications in appropriately selected patients to maximize the symptomatic relief from pruritus.

### Additional remarks

#### Polytherapy for postburn itch

Studies in the subject have concluded that a single agent is most likely to be insufficient in eradicating postburn itch. Hence, a combination of interventions is needed to achieve satisfactory relief in the majority of patients.[2,38] We advocate the judicious and stepwise use of agents in our protocols. Our clinical experience indicates that, in a limited number of patients, a single agent may be sufficient in controlling symptoms. An incomplete or absent response should warrant regimen escalation either by means of agent addition or substitution. We recommend that firstline agents are tried before second-line agents and that clinicians utilise interventions in all three categories in our protocols, namely peripherally, centrally acting agents and non- pharmacological adjuncts.

#### Choice between categories of intervention employed for postburn itch

Our protocols categorise therapeutic options according to the GRADE strength of recommendation (first vs. second line) and nature of intervention (pharmacological and adjuncts). The choice between peripherally and centrally acting agents needs careful consideration and calls upon consideration of the likely pathophysiological mechanisms underlying the postburn itch.

The pathophysiology of pruritus has been widely believed to be of peripheral origin, with histamine being the chief mediator for the generation of stimuli acting on primary neuronal afferents.[44] There is accumulating evidence of a central/neuropathic contribution in the generation and maintenance of pruritic symptoms, implying a role for sensitisation in the central nervous system;[45] nevertheless, explicit neuropathic mechanisms have not been formally proposed in the burns literature. The preliminary evidence regarding the superiority of gabapentin as monotherapy for burns pruritus may help to support the involvement of the central nervous system components in symptom generation and maintenance into a chronic state. Neuropathic phenomena are known to be of either acute or chronic nature and further research will be needed to clarify whether any possible neuropathic phenomena participate in burns itch pathophysiology and their exact timescales. Additionally, the ability of centrally acting agents to prevent any possible neuropathic phenomena related to the pruritic pathway needs to be further elucidated.

Recent evidence suggests the existence of two subtypes of pruritus:[46]

- Acute pruritus affecting the majority of patients and possibly relating to a period from wound closure to the early remodelling phase of healing, and
- Chronic pruritus affecting a subgroup of patients with deeper burns and early posttraumatic stress disorder (PTSD) symptoms.

The same work has identified an interesting pattern of risk factors at various times of rehabilitation. At 3 months, pruritic complaints were predicted by female sex, TBSA, number of surgical procedures undergone by the patient and PTSD symptoms as measured at 2 weeks postburn. At 12 months, female sex, number of surgical procedures and PTSD symptoms were significant predictors, whereas at 24 months, only the latter two variables persisted as predictors for postburn pruritus.

This study provides an excellent platform towards identifying patients most likely to develop chronic pruritus and allows clinicians to tailor regimens using the most appropriate agents. In the current state of knowledge, we advocate the early employment of centrally acting agents in combination with peripherally acting agents in all patients, especially in those at a high risk of chronic pruritus development.

## CONCLUSION

We have conducted a detailed review of all the available evidence on therapeutic interventions for postburn itch to date. It is apparent that most studies are of not sufficient statistical power to allow the recommendation of certain agents for the treatment of burns pruritus. We additionally present our approach to derive a treatment template for adult and paediatric patients using the GRADE classification, which is a widely accepted system to make recommendations in clinical practice. Validation of treatment protocols in the subject area is of paramount importance, but is currently hindered by the lack of research to define the ‘gold standard’ for treatment. We recommend the judicious use of a combination of peripherally and centrally acting pharmacological agents as well as the use of nonpharmacological interventions for the treatment of postburn itch.
